# Real-world effectiveness and safety of ibrutinib in patients with chronic lymphocytic leukemia in Japan: the Orbit study

**DOI:** 10.1007/s12185-024-03875-0

**Published:** 2024-11-26

**Authors:** Tsuyoshi Muta, Yosuke Masamoto, Go Yamamoto, Shingo Kurahashi, Yoshihiro Kameoka, Shuichi Ota, Eri Matsuki, Kazutaka Ozeki, Takanori Toyama, Naoki Takahashi, Takahiro Kumode, Nobuyuki Aotsuka, Takuro Yoshimura, Hideto Tamura, Ai Omi, Kazuhiro Shibayama, Aki Watanabe, Yasushi Isobe, Kensuke Kojima, Jun Takizawa, Hirokazu Nagai, Junji Suzumiya, Sadao Aoki

**Affiliations:** 1 Department of Transfusion Medicine Clinical Laboratory, Hiroshima Red Cross Hospital and Atomic-Bomb Survivors Hospital, Hiroshima, Japan; 2 Department of Cell Therapy and Transplantation Medicine, The University of Tokyo Hospital, Tokyo, Japan; 3 Department of Hematology, Toranomon Hospital, Tokyo, Japan; 4 Department of Hematology and Oncology, Toyohashi Municipal Hospital, Aichi, Japan; 5 Department of Hematology, Nephrology and Rheumatology, Akita University Graduate School of Medicine and Faculty of Medicine, Akita, Japan; 6 Department of Hematology, Akita Red Cross Hospital, Akita, Japan; 7 Department of Hematology, Sapporo Hokuyu Hospital, Sapporo, Japan; 8 Division of Hematology, Department of Medicine, Keio University School of Medicine, Tokyo, Japan; 9 Department of Hematology and Oncology, JA Aichi Konan Kosei Hospital, Aichi, Japan; 10 Department of Internal Medicine, Miyazaki Prefectural Nobeoka Hospital, Miyazaki, Japan; 11 Department of Hematology, International Medical Center, Saitama Medical University, Saitama, Japan; 12 Department of Hematology and Rheumatology, Kindai University Hospital, Osaka, Japan; 13 Department of Hematology and Oncology, Japanese Red Cross Narita Hospital, Chiba, Japan; 14 Department of Hematology, Osaka City General Hospital, Osaka, Japan; 15 Department of Diabetes, Endocrinology and Hematology, Dokkyo Medical University Saitama Medical Center, Saitama, Japan; 16 Department of Hematology, Nippon Medical School, Tokyo, Japan; 17 Department of Medical Affairs, Janssen Pharmaceutical K.K, 3-5-2 Nishi-Kanda, Chiyoda-Ku, Tokyo, 101-0065 Japan; 18 Statistics & Decision Sciences Japan, Janssen Pharmaceutical K.K., Tokyo, Japan; 19 Medical Affairs Delivery Unit, Global Development, Janssen Pharmaceutical K.K., Tokyo, Japan; 20 Department of Medical Oncology, Hematology and Infectious Diseases, Fukuoka University Hospital, Fukuoka, Japan; 21 Department of Hematology, Kochi Medical School, Kochi University, Kochi, Japan; 22 Department of Hematology, Endocrinology and Metabolism, Niigata University Faculty of Medicine, Niigata, Japan; 23 Hematology Department, National Hospital Organization Nagoya Medical Center, Aichi, Japan; 24 Department of Hematology, Koga Community Hospital, Yaizu, Japan; 25 Department of Medical Technology, Faculty of Medical Technology, Niigata University of Pharmacy and Medical and Life Sciences, Niigata, Japan; 26 Department of Hematology, Niigata Minami Hospital, Niigata, Japan

**Keywords:** Bruton’s tyrosine kinase inhibitor, Chronic lymphocytic leukemia, Ibrutinib, Japan, Real-world study, Small lymphocytic lymphoma

## Abstract

Ibrutinib is a first-in-class Bruton’s tyrosine kinase inhibitor that is approved for the treatment of chronic lymphocytic leukemia (CLL)/small lymphocytic lymphoma (SLL) in Japan based on randomized clinical trial data. The aim of the real-world, retrospective Orbit study was to describe long-term clinical outcomes and management in adults (aged ≥ 20 years) with CLL/SLL treated with ibrutinib, either as first-line (1L) treatment or for relapsed or refractory (RR) disease, in routine clinical practice in Japan between July 2018 and December 2020. A total of 246 patients were registered, and the safety and per-protocol sets included 237 and 234 patients, respectively. After a median follow-up of 35.7 months, the 36-month progression-free survival rate was 80.9% in the 1L CLL cohort and 67.2% in the RR CLL cohort, and the 36-month overall survival rates were 90.8% and 83.7%, respectively. Common Terminology Criteria for Adverse Events (CTCAE) Grade ≥ 3 adverse events of special interest were atrial fibrillation (2.1%), infections (herpesvirus infection, fungal infection, or *Pneumocystis jiroveci* pneumonia; 1.7%), bleeding (3.8%), and second primary malignancy (2.5%). These findings confirm the long-term, real-world effectiveness and safety of ibrutinib for the treatment of Japanese patients with newly diagnosed or RR CLL/SLL.

**Supplementary Information:**

The online version contains supplementary material available at 10.1007/s12185-024-03875-0.

## Introduction

Chronic lymphocytic leukemia (CLL) and small lymphocytic lymphoma (SLL), which are considered to be the same disease, are forms of non-Hodgkin lymphoma that are characterized by clonal proliferation and accumulation of small CD5-positive B cells [1]. Although common in Western countries [2], CLL/SLL is quite rare in Asian countries, including Japan, with an incidence of 0.2 per 100,000 in 2008 (vs 3.5 per 100,000 in the United States [US]) [3]. The epidemiology of CLL in Japan differs from that in Western countries, including a higher frequency of mutation in the immunoglobulin heavy chain variable region (*IGHV*) gene and lower frequency of 17p deletion compared with European populations [4, 5].

Ibrutinib, a first-in-class Bruton’s tyrosine kinase inhibitor, is approved for the treatment of patients with CLL/SLL in Japan [6]. Global phase 3 randomized controlled trials (RCTs) demonstrated the superior efficacy of single-agent ibrutinib in patients with CLL or SLL, with significantly improved progression-free survival (PFS) and overall survival (OS) compared with ofatumumab in patients with relapsed or refractory (RR) disease (RESONATE) [7] or chlorambucil in previously untreated patients (RESONATE-2) [8]. However, these RCTs [7, 8] and other large phase 3 RCTs of ibrutinib (vs zanubrutinib; ALPINE [9] or acalabrutinib; ELEVATE-RR [10]) did not enroll patients from Japan, and real-world evidence of ibrutinib use in Japanese patients with CLL/SLL is limited.

Post-marketing surveillance (PMS) in Japanese patients with RR CLL/SLL previously demonstrated that the effectiveness and safety of ibrutinib over 1 year in routine clinical practice was consistent with RCT data [11]. Given the limited follow-up of that PMS, there is a need for longer term, real-world data on the effectiveness and safety of ibrutinib in Japanese patients with CLL/SLL. Further clarification of the real-world management of adverse events of special interest (AESIs) during ibrutinib treatment is also needed, including atrial fibrillation (AF), infections, Grade ≥ 3 bleeding (as defined by Common Terminology Criteria for Adverse Events [CTCAE]), and second primary malignancy (SPM), which are known to occur with ibrutinib and other Bruton’s tyrosine kinase inhibitors [7, 8, 11, 12]. In addition, it is important to describe AESIs in Japanese patients because of differences in the epidemiology of cerebrovascular and cardiovascular diseases compared with their Western counterparts [13].

Therefore, the retrospective, observational Orbit study was conducted to describe longer-term real-world clinical outcomes and management of Japanese patients with CLL/SLL who initiated ibrutinib treatment, either as first-line (1L) treatment or as subsequent treatment in those with RR disease.

## Materials and methods

### Study design and patients

Orbit was a multicenter, retrospective, observational study of ibrutinib use in patients with CLL/SLL treated in routine clinical practice in Japan (as shown in Supplementary Figure S1). Adult patients (aged ≥ 20 years) with a confirmed diagnosis of CLL/SLL (in accordance with local practice) who initiated ibrutinib, either as 1L treatment (1L CLL cohort) or for RR disease (RR CLL cohort), from July 1, 2018 (the Japanese marketing authorization date) to December 31, 2020 were eligible for inclusion. Patients were excluded if they had any of the following: received ibrutinib without following the package insert instructions; other cancer at the time of ibrutinib initiation; experienced Richter’s syndrome before ibrutinib initiation; previously received ibrutinib; received an investigational drug or used an investigative invasive medical device during the study period; or been enrolled in another Janssen-sponsored study (including PMS) during the study period. Patients were treated in accordance with routine clinical practice in the outpatient specialist care setting in Japan, with their treating physician having previously made the decision to initiate ibrutinib treatment.

The protocol was approved by the central and/or local Ethics Review Committee for Ethical Guidelines before the start of the study, according to the regulations. All procedures were conducted according to the principles of the Declaration of Helsinki. Patients (or their legal representative) provided written informed consent before study enrollment. In cases where it was difficult to obtain informed consent (e.g., due to death or other reasons), opt-out enrollment was permitted.

### Data collection and outcomes

All data were extracted retrospectively from patients’ medical records from the initiation of ibrutinib through to the end of the follow-up period (limited historical data from the period prior to ibrutinib initiation were also extracted). Extracted data included patient baseline characteristics, CLL/SLL clinical and treatment history, Eastern Cooperative Oncology Group performance status (ECOG PS), and Cumulative Illness Rating Scale (CIRS) score (range 0–52, with higher scores indicating worse health status) [14]. Data related to ibrutinib treatment and subsequent patient management to avoid/minimize infection-related adverse events (AEs), treatment response, and safety were also extracted. The end of follow-up for each patient was the last data collection timepoint within the study, which was December 31, 2022 at the latest.

The primary endpoint was the 36-month rate of PFS in the 1L CLL cohort. PFS was defined as the time from the date of first dose of ibrutinib until the date of progressive disease (PD) or death from any cause, whichever came first. Secondary endpoints were the 36-month rate of PFS in the RR CLL cohort, and the OS, best response, and disease control rate (DCR; defined as the proportion of patients who achieved complete response [CR], partial response [PR], or stable disease) in the 1L CLL and RR CLL cohorts. The date of PD was defined as the physician’s assessment of the date of progression in accordance with routine clinical practice (e.g., the international workshop on Chronic Lymphocytic Leukemia [iwCLL] criteria [15]) from the start date of ibrutinib to the start date of the first post-ibrutinib anti-cancer therapy. Best response to ibrutinib treatment (CR, PR, stable disease, PD or unknown) were assessed by physicians in accordance with their routine clinical practice (e.g., the iwCLL criteria) from the date of the first dose of ibrutinib until the date on which ibrutinib treatment was stopped. Response to treatment was only recorded if the patient's medical records contained sufficient information to determine the therapeutic effect of ibrutinib or the therapeutic effect within the scope of routine medical care. Time to next treatment (TTNT) was assessed as an exploratory endpoint and was defined as the time from the date of first ibrutinib dose to the date of subsequent anticancer therapy for CLL or death from any cause.

AESIs during ibrutinib treatment were recorded and graded according to CTCAE version 5.0, and included AF, infections (i.e., herpesvirus infections, fungal infections, and *Pneumocystis jiroveci* pneumonia [PJP]), Grade ≥ 3 bleeding events, and SPMs. An AESI was considered serious if it: resulted in death; was life-threatening; required inpatient hospitalization or prolongation of existing hospitalization; resulted in persistent or significant disability/incapacity; was a congenital anomaly/birth defect; was a suspected transmission of any infectious agent via a medicinal product; or was considered to be medically important (i.e., might require intervention to prevent one of the other outcomes listed above).

### Statistical analysis

As this was a descriptive study without formal hypothesis testing, calculation of sample size with statistical power was not conducted. The retrospective chart review aimed to enroll approximately 200 patients.

The effectiveness analyses were conducted in the per-protocol set (defined as all patients enrolled in the study who received ibrutinib and met the eligibility criteria) separately for the 1L CLL and RR CLL cohorts. The safety profile of ibrutinib was assessed in the safety analysis set (defined as all patients enrolled in the study who received ibrutinib).

Categorical variables were summarized as the number and proportion (with two-sided 95% confidence interval [CI]), and continuous variables were reported as mean, standard deviation, median, and range (minimum–maximum). For all time-to-event variables, including PFS, OS, and TTNT, the Kaplan–Meier method was used to estimate median survival rates at selected timepoints with 95% CI, with missing data imputed.

All statistical analyses were conducted using SAS software, version 9.4 (SAS Institute, Cary, North Carolina, USA).

## Results

### Study population

**Fig. 1:**
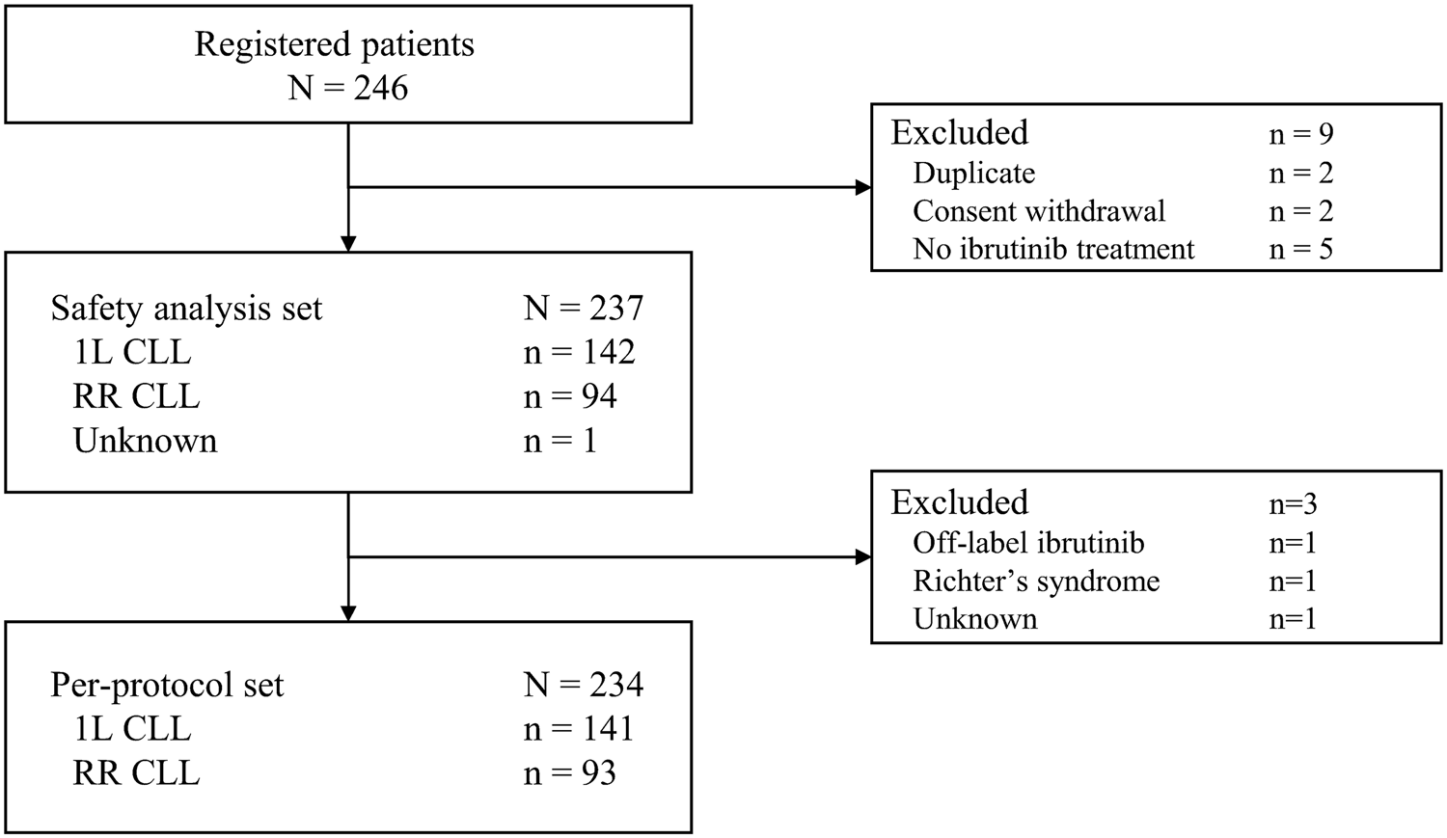
Patient flow diagram. *1L* first-line, *CLL* chronic lymphocytic leukemia, *RR* relapsed or refractory

In total, 246 patients were registered from 61 participating institutions, of whom 237 were included in the safety analysis set and 234 were included in the per-protocol set (Fig. 1). In the safety analysis set, 209 patients (88.2%) had CLL and 27 (11.4%) had SLL; CLL/SLL status was unknown for one patient, for whom no data was collected as exclusion criteria were met immediately after registration (Table 1). Ibrutinib was administered as 1L treatment in 142 patients (59.9%) and for RR disease in 94 patients (39.7%). Patients had a median age of 73 years, 60.8% of patients were male, and 63.3% were aged ≥ 70 years. ECOG PS was ≥ 2 in 5.5% of patients and CIRS score was ≥ 6 in 13.5%. Among patients with CLL, Rai stage was III in 18.2% of patients and IV in 23.0%, and 38.3% had Binet stage C disease. The mutation (e.g., *IGHV*) and 17p deletion status were rarely evaluated (data not shown).

**Table 1:** Patient baseline characteristics in the safety analysis set

	Total population^a^ n = 237	1L CLL n = 142	RR CLL n = 94
Male sex, n (%)	144 (60.8)	87 (61.3)	57 (60.6)
Age, years, median (range)	73 (28–90)	72 (34–90)	74 (28–89)
Age ≥ 70 years, n (%)	150 (63.3)	86 (60.6)	64 (68.1)
ECOG PS, n (%)			
0	128 (54.0)	82 (57.7)	46 (48.9)
1	57 (24.1)	35 (24.6)	22 (23.4)
2	11 (4.6)	5 (3.5)	6 (6.4)
3	1 (0.4)	1 (0.7)	0
4	1 (0.4)	1 (0.7)	0
Unknown	39 (16.5)^a^	18 (12.7)	20 (21.3)
CIRS score			
Mean ± SD	3.2 ± 3.3	3.0 ± 3.4	3.6 ± 3.2
Category ≥ 6, n (%)	32 (13.5)	17 (12.0)	15 (16.0)
Unknown	68 (28.7)^a^	40 (28.2)	27 (28.7)
Diagnosis, n (%)			
CLL	209 (88.2)^a^	127 (89.4)	82 (87.2)
Rai stage	n = 209	n = 127	n = 82
0	18 (8.6)	14 (11.0)	4 (4.9)
I	20 (9.6)	13 (10.2)	7 (8.5)
II	34 (16.3)	21 (16.5)	13 (15.9)
III	38 (18.2)	25 (19.7)	13 (15.9)
IV	48 (23.0)	29 (22.8)	19 (23.2)
Unknown	51 (24.4)	25 (19.7)	26 (31.7)
Binet stage	n = 209	n = 127	n = 82
A	28 (13.4)	20 (15.7)	8 (9.8)
B	46 (22.0)	28 (22.0)	18 (22.0)
C	80 (38.3)	51 (40.2)	29 (35.4)
Unknown	55 (26.3)	28 (22.0)	27 (32.9)
SLL	27 (11.4)^a^	15 (10.6)	12 (12.8)
Ann Arbor Stage	n = 27	n = 15	n = 12
I	1 (3.7)	1 (6.7)	0
II	4 (14.8)	0	4 (33.3)
III	3 (11.1)	1 (6.7)	2 (16.7)
IV	14 (51.9)	10 (66.7)	4 (33.3)
Unknown	5 (18.5)	3 (20.0)	2 (16.7)
Symptomatic classification	n = 27	n = 15	n = 12
A	19 (70.4)	10 (66.7)	9 (75.0)
B	4 (14.8)	2 (13.3)	2 (16.7)
Unknown diagnosis	4 (14.8)	3 (20.0)	1 (8.3)

1L, first-line; CIRS, Cumulative Illness Rating Scale; CLL, chronic lymphocytic leukemia; ECOG PS, Eastern Cooperative Oncology Group performance status; RR, relapsed or refractory; SD, standard deviation; SLL, small lymphocytic lymphoma

^a^One patient, who had unknown CLL/SLL status, met exclusion criteria immediately after registration and had no data collected thereafter

In the RR CLL cohort, patients had received a median (range) of 2 (1–6) prior treatment lines (as shown in Supplementary Table S1). The most common prior regimens (in ≥ 10% of patients) were fludarabine (23.4%), cyclophosphamide (13.8%), and bendamustine + rituximab (10.6%).

The baseline laboratory values, including immunoglobulin (Ig) A, IgM, and IgG levels, white blood cell counts, and neutrophil counts are summarized in Supplementary Table S2.

### Ibrutinib treatment

After a median follow-up period of 35.7 months, ibrutinib treatment was ongoing in 124 patients (53.0%) and discontinued in 110 (47.0%; Table 2). The most common reason for ibrutinib discontinuation was AE occurrence (n = 52; 22.2%), followed by PD (n = 31; 13.2%). At the end of the observation period, 31 patients (13.2%) had died; none of these deaths were attributed to ibrutinib treatment.

**Table 2:** Treatment disposition at the end of the observation period in the per-protocol set

n (%)	Total population, n = 234	1L CLL, n = 141	RR CLL, n = 93
Continuing ibrutinib at study end	124 (53.0)	85 (60.3)	39 (41.9)
Discontinued ibrutinib	110 (47.0)	56 (39.7)	54 (58.1)
AE	52 (22.2)	28 (19.9)	24 (25.8)
Disease progression	31 (13.2)	16 (11.3)	15 (16.1)
Death	7 (3.0)	6 (4.3)	1 (1.1)
Other^a^	20 (8.5)	6 (4.3)	14 (15.1)

*1L* first-line, *AE* adverse event, *CLL* chronic lymphocytic leukemia, *RR* relapsed or refractory

^a^‘Other’ reasons included avoidance of AEs, patient request, transfer to hospital, and physician’s decision

In the safety analysis set, the median (range) duration of treatment (DoT) for ibrutinib was 30.6 (0.2–53.9) months in the total population, 31.5 (0.8–53.9) months in the 1L CLL cohort and 28.3 (0.2–53.3) months in the RR CLL cohort. The median (range) relative dose intensity was 97.6% (6.2–100.0) in the total population, and 97.7% (8.9–100.0) and 97.9% (6.2–100.0) in the respective cohorts.

The initial ibrutinib dose was reduced in 27.0% of patients in the total population (25.4% in the 1L CLL cohort and 29.8% in the RR CLL cohort). Of those who had initial ibrutinib dose reduction, the main reason for this was AE avoidance (in 51 of 64 patients [79.7%]).

### Effectiveness

**Fig. 2:**
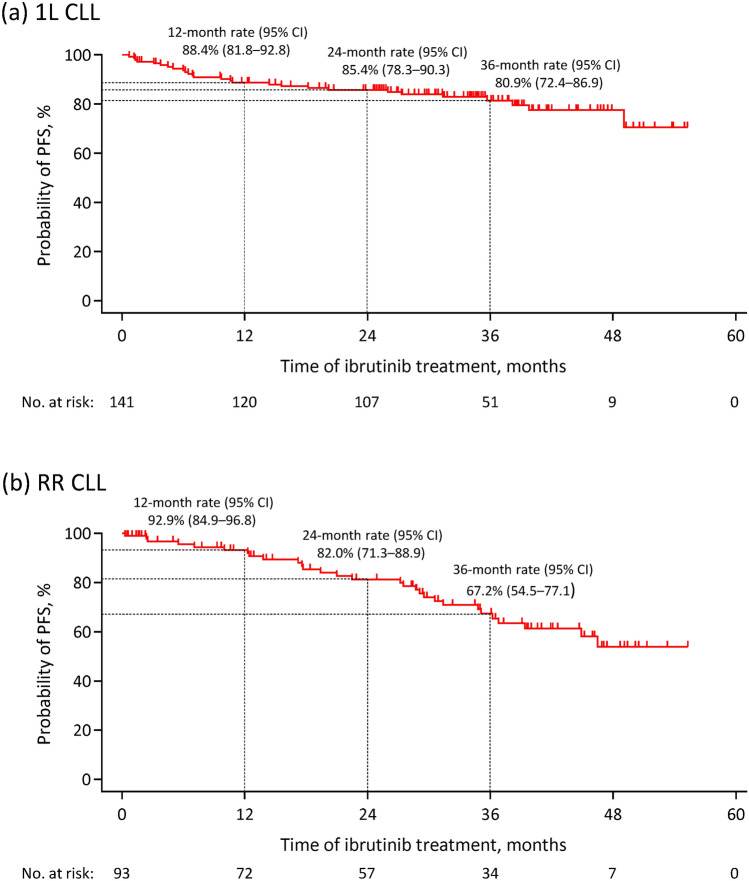
Kaplan–Meier curves of progression-free survival in patients with chronic lymphocytic leukemia receiving ibrutinib as **a** first-line therapy and **b** for relapsed or refractory disease in the per-protocol set. *1L* first-line, *CI* confidence interval, *CLL* chronic lymphocytic leukemia, *No.* number, *PFS* progression-free survival, *RR* relapsed or refractory

The 36-month rate of PFS was 80.9% (95% CI 72.4–86.9) in the 1L CLL cohort (Fig. 2a) and 67.2% (95% CI 54.5–77.1) in the RR CLL cohort (Fig. 2b). At 12 months, the PFS rate was 88.4% (95% CI 81.8–92.8) and 92.9% (95% CI 84.9–96.8) in the respective cohorts. At 24 months, the PFS rate was 85.4% (95% CI 78.3–90.3) and 82.0% (95% CI 71.3–88.9) in the respective cohorts.

**Fig. 3:**
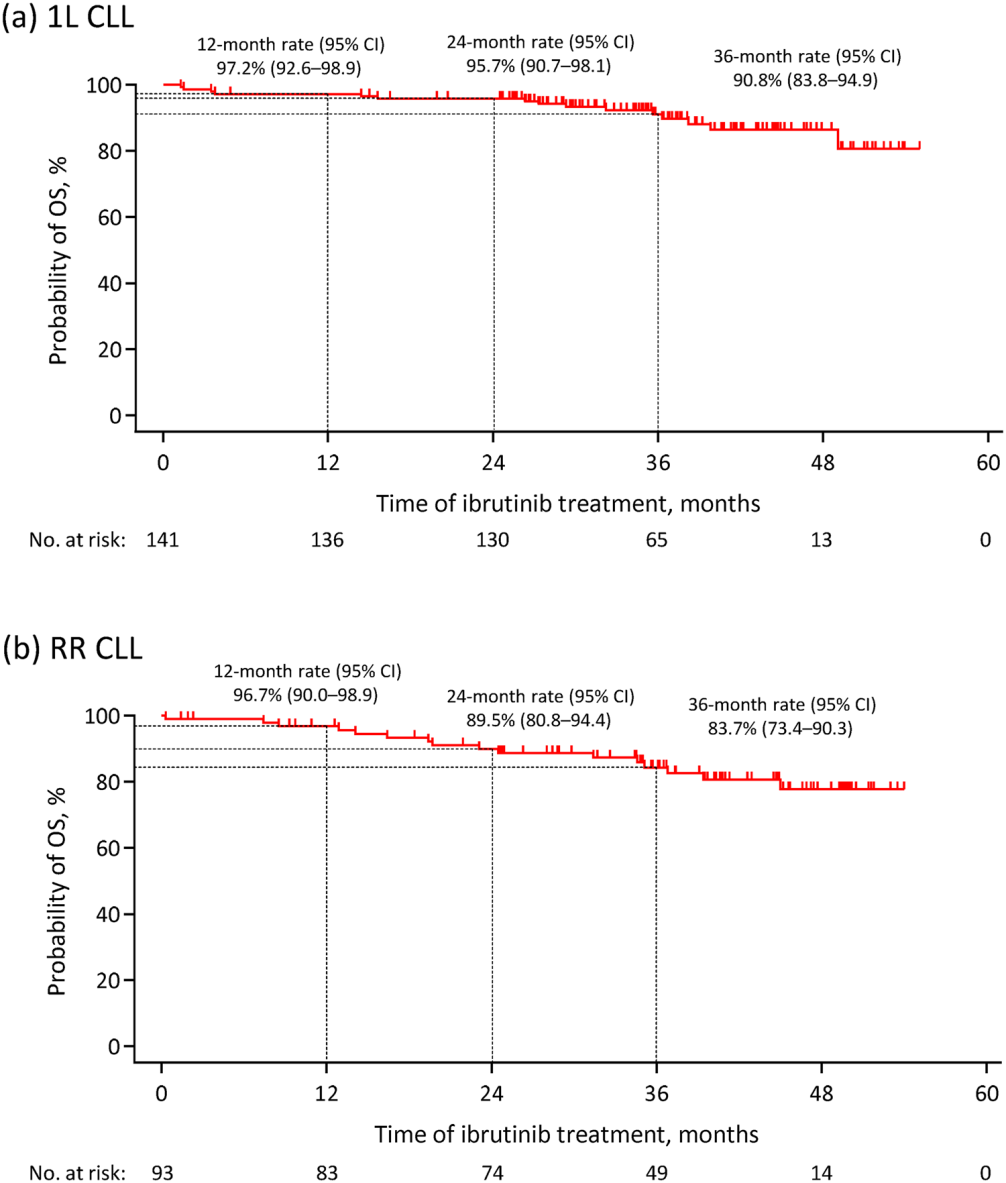
Kaplan–Meier curves of overall survival in patients with chronic lymphocytic leukemia receiving ibrutinib as **a** first-line therapy and **b** for relapsed or refractory disease in the per-protocol set. *1L* first-line, *CI* confidence interval, *CLL* chronic lymphocytic leukemia, *No.* number, *OS* overall survival, *RR* relapsed or refractory

The OS rates at 12, 24, and 36 months were 97.2% (95% CI 92.6–98.9), 95.7% (95% CI 90.7–98.1), and 90.8% (95% CI 83.8–94.9), respectively, in the 1L CLL cohort (Fig. 3a). The OS rates at 12, 24, and 36 months were 96.7% (95% CI 90.0–98.9), 89.5% (95% CI 80.8–94.4), and 83.7% (95% CI 73.4–90.3), respectively, in the RR CLL cohort (Fig. 3b).

The ORR was 76.6% (95% CI 66.7–84.7) in the 1L CLL cohort and 68.5% (95% CI 54.4–80.5) in the RR CLL cohort (Table 3). In the 1L CLL cohort, 19 patients (20.0%) achieved CR and 53 (55.8%) achieved PR. CR and PR were achieved by nine (16.4%) and 28 (50.9%) patients in the RR CLL cohort. The DCR was 90.4% (95% CI 82.6–95.5) and 96.3% (87.3–99.5) in the respective cohorts.

**Table 3:** Best response with ibrutinib treatment (per-protocol set)

	1L CLL, n = 95	RR CLL, n = 55
Best response, n (%)		
CR	19 (20.0)	9 (16.4)
PR	53 (55.8)	28 (50.9)
SD	13 (13.7)	15 (27.3)
PD	9 (9.5)	2 (3.6)
NE	1 (1.1)	1 (1.8)
ORR,^a^ % (95% CI)	76.6 (66.7–84.7)	68.5 (54.4–80.5)
DCR,^b^ % (95% CI)	90.4 (82.6–95.5)	96.3 (87.3–99.5)

*1L* first-line, *CI* confidence interval, *CLL* chronic lymphocytic leukemia, *CR* complete response, *DCR* disease control rate, *NE* not evaluable, *ORR* objective response rate, *PD* progressive disease, *PR* partial response, *RR* relapsed or refractory, *SD* stable disease

^a^Calculated using the following formula: (CR + PR)/(CR + PR + SD + PD) × 100

^b^Calculated using the following formula: (CR + PR + SD)/(CR + PR + SD + PD) × 100

The 36-month TTNT rate was 73.7% (95% CI 65.0–80.5) in the 1L CLL cohort (as shown in Supplementary Figure S2a) and 56.9% (95% CI 45.1–67.1) in the RR CLL cohort (as shown in Supplementary Figure S2b).

Subsequent anti-CLL treatment was administered in 31 patients (22.0%) in the 1L CLL cohort and 31 patients (33.3%) in the RR CLL cohort (as shown in Supplementary Table S3). In the 1L CLL cohort, most of these patients received treatment with venetoclax ± rituximab (38.7%) or acalabrutinib (32.3%), and in the RR CLL cohort, the most common post-ibrutinib regimens were venetoclax ± rituximab (45.2%), cyclophosphamide (12.9%), or rituximab, cyclophosphamide, doxorubicin, vincristine, and prednisone (R-CHOP; 9.7%).

### Safety

In the safety analysis set, any grade AESIs were reported in 48 patients (20.3%) in the total population, 32 patients (22.5%) in the 1L CLL cohort and 16 (17.0%) in the RR CLL cohort (Table 4).

**Table 4:** Adverse events of special interest during ibrutinib treatment in the safety analysis set

AESI, n (%)	Total population, n = 237^a^		1L CLL, n = 142		RR CLL, n = 94	
	Any grade	Grade ≥ 3	Any grade	Grade ≥ 3	Any grade	Grade ≥ 3
Any AESI	48 (20.3)		32 (22.5)		16 (17.0)	
AF	21 (8.9)	5 (2.1)	14 (9.9)	3 (2.1)	7 (7.4)	2 (2.1)
Infections	12 (5.1)^b^	4 (1.7)	9 (6.3)^b^	4 (2.8)	3 (3.2)	
Herpesvirus infection	6 (2.5)	3 (1.3)	5 (3.5)	3 (2.1)	1 (1.1)	0
Fungal infection	5 (2.1)	0	3 (2.1)	0	2 (2.1)	0
PJP	2 (0.8)	1 (0.4)	2 (1.4)	1 (0.7)	0	0
Bleeding^c^		9 (3.8)		4 (2.8)		5 (5.3)
SPM	9 (3.8)	6 (2.5)	6 (4.2)	4 (2.8)	3 (3.2)	2 (2.1)

*1L* first-line, *AESI* adverse event of special interest, *AF* atrial fibrillation, *CLL* chronic lymphocytic leukemia, *PJP* *Pneumocystis jiroveci* pneumonia, *RR* relapsed or refractory, *SPM* secondary primary malignancy

^a^Includes one patient with unknown CLL/SLL status, who met exclusion criteria immediately after registration and had no data collected thereafter

^b^Includes two patients who each had two different infections of interest (one with two fungal infections and the other with a herpesvirus infection and a fungal infection)

^c^Grade ≥ 3 bleeding events extracted from the medical records included cerebral hemorrhage, epistaxis, gastrointestinal hemorrhage, hemorrhage subcutaneous melena, oral mucosa hemorrhage, pericardial hemorrhage, subcutaneous hematoma, subdural hematoma, or subdural hemorrhage

Any grade AF was reported in 21 patients (8.9%) during ibrutinib treatment and was considered to be drug-related in 18 patients (7.6%; Table 5). None of the 21 patients had a prior history of AF. Of the 21 patients with AF, five patients (2.1%) required hospitalization/prolonged hospitalization for this AE. Grade ≥ 3 AF was reported in five patients (2.1%). Ibrutinib treatment could be continued without dose modification in 11 patients (4.6%), while ibrutinib was interrupted in four patients (1.7%) and discontinued in six patients (2.5%). Medications for AF were administered in 14 patients (5.9%), including anticoagulants, β-blockers, calcium channel blockers, antiarrhythmics, and other.

**Table 5:** List of individual patients with atrial fibrillation during ibrutinib treatment

Patient no	Prior history of AF	Time to AE onset, months	Seriousness^a^	CTCAE Grade	Causal relationship with ibrutinib	Change in ibrutinib treatment^b^	Outcome
1L CLL							
1	No	17.9	Not serious	2	Yes	Discontinued	Not recovered
2	No	0.6	Not serious	1	Yes	No change	Complete recovery
3	No	16.7	Not serious	2	Yes	No change	Not recovered
4	No	6.9	Not serious	2	Yes	No change	Not recovered
5	No	18.4	Not serious	1	Yes	Discontinued	Partial recovery
6	No	8.3	Not serious	2	Yes	Discontinued	Partial recovery
7	No	0.9	Not serious	3	Yes	Interrupted	Partial recovery
8	No	3.6	Not serious	2	Yes	No change	Not recovered
9	No	25.1	Not serious	2	Yes	Discontinued	Complete recovery
10	No	28.4	Not serious	2	Yes	No change	Complete recovery
11	No	5.9	Serious	2	Yes	No change	Complete recovery
12	No	19.7	Not serious	2	No	No change	Partial recovery
13	No	9.6	Serious	3	Yes	No change	Partial recovery
14	No	1.8	Serious	3	Yes	Discontinued	Unknown
RR CLL							
15	No	1.0	Not serious	3	Yes	No change	Complete recovery
16	No	12.3	Not serious	2	Yes	Discontinued	Partial recovery
17	No	9.2	Serious	2	Yes	Interrupted	Partial recovery
18	No	31.8	Not serious	3	No	No change	Complete recovery
19	No	22.4	Not serious	1	Yes	Interrupted	Partial recovery
20	No	11.4	Not serious	2	No	No change	Partial recovery
21^c^	No	4.6	Serious	1	Yes	Interrupted	Partial recovery
		6.8	Serious	1	Yes	Interrupted	Partial recovery
		12.9	Not serious	1	Yes	Interrupted	Complete recovery

*1L* first-line, *AE* adverse event, *AF* atrial fibrillation, *CLL* chronic lymphocytic leukemia, *CTCAE* Common Terminology Criteria for Adverse Events, *RR* relapsed or refractory

^a^Serious events were those that: resulted in death; were life-threatening; required inpatient hospitalization or prolongation of existing hospitalization; resulted in persistent or significant disability/incapacity; was a congenital anomaly/birth defect; was a suspected transmission of any infectious agent via a medicinal product; or was considered to be medically important

^b^No change = no dose adjustment; interrupted = dose hold and restart

^c^This patient experienced three AF events

In the safety analysis set, herpesvirus infection, fungal infection, or PJP occurred in 12 patients (5.1%; Table 4). None of these infections led to death. Antimicrobial prophylaxis for herpesvirus infection, fungal infection, and PJP was administered in 77 (32.5%), 21 (8.9%), and 99 (41.8%) patients, respectively, of whom none developed these infections (Table 6). Of the 12 patients who developed infections (herpesvirus infection [n = 6; 2.5%], fungal infection [n = 5; 2.1%], and PJP [n = 2; 0.8%]), none had received antimicrobial prophylaxis. Two of these patients had two different infections of interest.

**Table 6:** Incidence of herpesvirus, fungal, and pneumocystis infections during ibrutinib treatment according to the use of antimicrobial prophylaxis in the safety analysis set

Infection, n (%)	Total population, n = 237^a^		1L CLL, n = 142		RR CLL, n = 94	
	With prophylaxis	Without prophylaxis	With prophylaxis	Without prophylaxis	With prophylaxis	Without prophylaxis
Herpesvirus	n = 77	n = 160	n = 45	n = 97	n = 32	n = 62
Incidence	0	6 (3.8)	0	5 (5.2)	0	1 (1.6)
Fungal	n = 21	n = 216	n = 13	n = 129	n = 8	n = 86
Incidence	0	5 (2.3)	0	3 (2.3)	0	2 (2.3)
Pneumocystis	n = 99	n = 138	n = 56	n = 86	n = 43	n = 51
Incidence	0	2 (1.4)	0	2 (2.3)	0	0

*1L* first-line, *CLL* chronic lymphocytic leukemia, *RR* relapsed or refractory

^a^Includes one patient with unknown CLL/SLL status, who met exclusion criteria immediately after registration and had no data collected thereafter

Grade ≥ 3 bleeding events were reported in nine patients (3.8%) in the total population; four (2.8%) in the 1L CLL cohort and five (5.3%) in the RR CLL cohort (Table 4). These Grade ≥ 3 bleeding events were serious gastrointestinal hemorrhage and non-serious epistaxis in two patients (0.8%) each, serious cerebral hemorrhage, pericardial hemorrhage, and subdural hemorrhage in one patient (0.4%) each, and non-serious melena and subcutaneous hematoma in one patient (0.4%) each. These patients were all recovering or had recovered within 4–29 days; there were no cases of repeated Grade ≥ 3 bleeding. In the total population, five patients were receiving anticoagulants and one was receiving antiplatelets during ibrutinib treatment; none of these patients experienced Grade ≥ 3 bleeding.

SPMs developed in nine patients (3.8%) in the safety analysis set (Table 7). These were colon and prostate cancer in two patients (0.8%) each, and breast cancer, gastric cancer, pancreatic carcinoma, sarcoma uterus, and invasive ductal breast carcinoma in one patient (0.4%) each. One SPM (colon cancer) was considered to be related to ibrutinib treatment. Three patients died due to SPM (colon cancer, pancreatic carcinoma, and sarcoma uterus).

**Table 7:** Second primary malignancies during ibrutinib treatment in the safety analysis set

n (%)	Total population, n = 237^a^	1L CLL, n = 142	RR CLL, n = 94
SPM	9 (3.8)	6 (4.2)	3 (3.2)
Grade 3	4 (1.7)	3 (2.1)	1 (1.1)
Grade 4	2 (0.8)	1 (0.7)	1 (1.1)
Grade 5	3 (1.3)	2 (1.4)	1 (1.1)
Type of SPM			
Breast cancer	1 (0.4)	1 (0.7)	0
Colon cancer	2 (0.8)	2 (1.4)	0
Gastric cancer	1 (0.4)	1 (0.7)	0
Pancreatic carcinoma	1 (0.4)	0	1 (1.1)
Sarcoma uterus	1 (0.4)	1 (0.7)	0
Prostate cancer	2 (0.8)	1 (0.7)	1 (1.1)
Invasive ductal breast carcinoma	1 (0.4)	0	1 (1.1)
Outcome			
Partial recovery	2 (0.8)	1 (0.7)	1 (1.1)
Complete recovery	1 (0.4)	0	1 (1.1)
Not recovered	2 (0.8)	2 (1.4)	0
Death	3 (1.3)	2 (1.4)	1 (1.1)
Unknown	1 (0.4)	1 (0.7)	0

*1L* first-line, *CLL* chronic lymphocytic leukemia, *RR* relapsed or refractory, *SPM* second primary malignancy

^a^Includes one patient with unknown CLL/SLL status, who met exclusion criteria immediately after registration and had no data collected thereafter

## Discussion

The retrospective Orbit study is the first to describe the longer-term real-world effectiveness and safety of ibrutinib treatment in Japanese patients with CLL/SLL. Ibrutinib demonstrated real-world effectiveness as a 1L-treatment for patients with CLL/SLL and as a treatment for previously treated patients with RR CLL/SLL. In addition, ibrutinib treatment had a manageable safety profile in both patient cohorts. Notably, Orbit is one of the largest observational studies of Japanese patients with CLL/SLL, and of the longest duration (median follow-up of 35.7 months vs 11.3 months for the prior Japanese PMS in patients with RR CLL/SLL [11]).

It is worth noting that some characteristics differ between the patients in our 1L CLL cohort and CLL/SLL populations in previously reported RCTs. For example, compared with the patients in RESONATE-2 (i.e., those who had previously untreated CLL/SLL), our 1L CLL cohort had a lower proportion of patients with ECOG PS of 2 (3.5% vs 8.6%) and CIRS ≥ 6 (12.0% vs 32.0% [CIRS > 6]) [8]. Similarly, compared with the RESONATE study population (patients with RR CLL/SLL), the RR CLL cohort of the current study had a lower proportion of patients with CIRS ≥ 6 (16.0% vs 32.0% [CIRS > 6]) [7]. The majority of patients in the RR CLL cohort had an ECOG PS 0–1 (72.3%); RESONATE exclusively enrolled patients with an ECOG PS of 0–1 [7].

The current study found that the real-world effectiveness of ibrutinib treatment in the 1L CLL cohort was consistent with that reported in the RESONATE-2 RCT in previously untreated patients, with 24 and 36-month PFS rates of 85.4% and 80.9% in Orbit versus 89% at 24 months in RESONATE-2 and 24-month OS rates of 95.7% versus 95% [16]. RESONATE-2 specifically excluded patients with the high-risk genomic feature of 17p deletion [8], and yet survival outcomes observed in the 1L CLL cohort in the current study were similar to RESONATE-2, even though some patients with this deletion may have been included the 1L CLL cohort. In the RR CLL cohort in the current study, the 36-month PFS rate (67.2%) was slightly higher than that reported in the RESONATE patient population of individuals with RR CLL/SLL (59%) [17]. Interestingly, the 24-month PFS rate in the RR CLL cohort (82.0%) was higher than that reported in the ibrutinib arm (65.9%) of the phase 3 ALPINE RCT of zanubrutinib versus ibrutinib in patients with RR CLL/SLL [9]. In the current study, genetic abnormalities were unknown. However, the epidemiology of CLL in Japan differs from that in Western countries, including a higher frequency of *IGHV* gene mutations and lower frequency of 17p deletion compared with European populations [4, 5], which explains at least in part the differences in survival outcomes between Orbit and the multinational studies ALPINE and RESONATE [9, 17].

In the RR CLL cohort of the current study, the 12-month PFS and OS rates (92.9% and 96.7%) were higher than in the previous Japanese PMS of ibrutinib in RR CLL/SLL (71.7% and 79.1%, respectively) [11]. This is likely because the number of prior treatment lines was smaller.

The ORR in the 1L CLL (76.6%) and RR CLL (68.5%) cohorts of the current study were lower than those reported in the RESONATE-2 (92%) [16] and RESONATE (91%) [17] RCTs. This could be due to some patients in the current real-world study not undergoing regular assessment of response or receiving subsequent treatment without any response assessment. Furthermore, the DCR in the current study exceeded 90% in both cohorts, indicating that most patients achieved adequate disease control with ibrutinib.

AESIs such as AF, infections, Grade ≥ 3 bleeding, and SPM, are a known class effect of Bruton’s tyrosine kinase inhibitors [7, 8, 11, 12]. With regard to the AESIs in our study, the incidence of any grade AF was similar between the 1L CLL and RR CLL cohorts (9.9% vs 7.4%), similar to the retrospective cohorts of the real-world studies (7.2–8.1%) [18, 19], and no higher than that reported during long-term follow-up of the RESONATE-2 RCT (10%; median ibrutinib DoT 28.5 months) [16], RESONATE (11%; median follow-up 44 months) [17], and ELEVATE-RR (16.0%; median follow-up 40.9 months) [10, 20]. The relatively low incidence of any-grade AF in the current study (8.9% of all patients) is notable given that patients in Orbit were elderly (median age 73 years). It should also be acknowledged that the prevalence [21], and possibly also the severity of AF, is generally lower in Japanese individuals than in Western populations. Only 2.1% of patients had Grade ≥ 3 AF in the current study. In addition, none of the 21 patients who developed this AESI during ibrutinib treatment had a history of AF. Patients undergoing ibrutinib treatment should be fully educated to be aware of changes in their heart rate regardless of their medical history.

In the current study, herpesvirus infection, fungal infection, and PJP had a low frequency (2.5%, 2.1%, and 0.8%, respectively), which was similar to that previously reported [11, 22, 23]. Although there is currently no international consensus regarding the use of antimicrobial prophylaxis during Bruton’s tyrosine kinase inhibitor therapy, in the current study, prophylaxis against herpesvirus infection, fungal infection, and PJP was administered in 32.5%, 8.9%, and 41.8% of patients, respectively. This could have been influenced by the high proportion of patients who were aged ≥ 70 years (63.3%) and also by the use of prior chemoimmunotherapy since PJP prophylaxis was more common in the RR CLL cohort. None of the patients who received prophylactic medications developed herpesvirus infection, fungal infection, or PJP. Similarly, a previous US study found that there were no cases of PJP during treatment containing either ibrutinib or acalabrutinib among patients who received PJP prophylaxis [22]. Although only patients without antimicrobial prophylaxis reported these infections in our study, antimicrobial prophylaxis may need to be considered in patients receiving Bruton’s tyrosine kinase inhibitors in the context of the individual patient’s infection risk.

The incidence of Grade ≥ 3 bleeding during ibrutinib treatment was 3.8% in the current study. None of the nine patients with Grade ≥ 3 bleeding were receiving concomitant anticoagulants or antiplatelets, and all were recovering or had recovered within a short period of time. These findings indicate that patients with CLL/SLL who have a high risk of bleeding are being managed appropriately by avoiding concomitant anticoagulants and antiplatelets and by interrupting/discontinuing ibrutinib when Grade ≥ 3 bleeding occurs.

There is a high risk of SPM, including melanoma and non-melanoma skin cancer, among patients with CLL/SLL [24]. However, the incidence of SPM in the current study was low (3.8%) and their relationship to ibrutinib treatment was unclear. There were no cases of skin cancer, although the incidence of skin cancer is considerably lower among Japanese than global populations [25].

The limitations of this study are inherent to those of retrospective, noninterventional studies, including the fact that only data that were available in the patients’ medical records were collected. Further, the patients’ response to ibrutinib treatment was not determined at a prespecified time point.

## Conclusions

In this real-world study of Japanese patients with CLL/SLL, the effectiveness of ibrutinib as 1L treatment or for RR disease was consistent with or better than that reported in global RCTs and other real-world studies. No new concerns were identified regarding AESIs, which could be managed appropriately according to the individual patient’s condition. To our knowledge, this is the largest, long-term, real-world study of the effectiveness and safety of ibrutinib for the treatment of CLL/SLL in Japanese patients; the results add to the wealth of data supporting the use of ibrutinib in this indication. The results of this study may serve as a useful historical control for the future evaluation of novel agents for CLL in Japan.

## Supplementary Information

Below is the link to the electronic supplementary material.

Supplementary file1 (DOCX 96 KB)

## Data Availability

The datasets generated and/or analyzed during the current study are not publicly available due to confidentiality clauses signed within the participating medical institutes.

## References

[1] Hallek M, Al-Sawaf O. Chronic lymphocytic leukemia: 2022 update on diagnostic and therapeutic procedures. Am J Hematol. 2021;96(12):1679–705. 10.1002/ajh.26367. 34625994 10.1002/ajh.26367

[2] Eichhorst B, Robak T, Montserrat E, Ghia P, Hillmen P, Hallek M, et al. Chronic lymphocytic leukaemia: ESMO Clinical Practice Guidelines for diagnosis, treatment and follow-up. Ann Oncol. 2015;26(Suppl 5):v78-84. 10.1093/annonc/mdv303. 26314781 10.1093/annonc/mdv303

[3] Chihara D, Ito H, Matsuda T, Shibata A, Katsumi A, Nakamura S, et al. Differences in incidence and trends of haematological malignancies in Japan and the United States. Br J Haematol. 2014;164(4):536–45. 10.1111/bjh.12659. 24245986 10.1111/bjh.12659 PMC3907701

[4] Takizawa J, Suzuki R, Izutsu K, Kiguchi T, Asaoku H, Saburi Y, et al. Characteristics of chronic lymphocytic leukemia in Japan: comprehensive analysis of the CLLRSG-01 study. Int J Hematol. 2024;119(6):686–96. 10.1007/s12185-024-03741-z. 38492198 10.1007/s12185-024-03741-z

[5] Tomomatsu J, Isobe Y, Oshimi K, Tabe Y, Ishii K, Noguchi M, et al. Chronic lymphocytic leukemia in a Japanese population: varied immunophenotypic profile, distinctive usage of frequently mutated IGH gene, and indolent clinical behavior. Leuk Lymphoma. 2010;51(12):2230–9. 10.3109/10428194.2010.527403. 21067444 10.3109/10428194.2010.527403

[6] Janssen Pharmaceutical K.K. Imbruvica® (ibrutinib): Japanese prescribing information [in Japanese]. Tokyo, Japan. 2023. https://www.pmda.go.jp/PmdaSearch/iyakuDetail/ResultDataSetPDF/800155_4291043M1027_1_15. Accessed 16 Apr 2024.

[7] Byrd JC, Brown JR, O’Brien S, Barrientos JC, Kay NE, Reddy NM, et al. Ibrutinib versus ofatumumab in previously treated chronic lymphoid leukemia. N Engl J Med. 2014;371(3):213–23. 10.1056/NEJMoa1400376. 24881631 10.1056/NEJMoa1400376 PMC4134521

[8] Burger JA, Tedeschi A, Barr PM, Robak T, Owen C, Ghia P, et al. Ibrutinib as initial therapy for patients with chronic lymphocytic leukemia. N Engl J Med. 2015;373(25):2425–37. 10.1056/NEJMoa1509388. 26639149 10.1056/NEJMoa1509388 PMC4722809

[9] Brown JR, Eichhorst B, Hillmen P, Jurczak W, Kazmierczak M, Lamanna N, et al. Zanubrutinib or ibrutinib in relapsed or refractory chronic lymphocytic leukemia. N Engl J Med. 2023;388(4):319–32. 10.1056/NEJMoa2211582. 36511784 10.1056/NEJMoa2211582

[10] Byrd JC, Hillmen P, Ghia P, Kater AP, Chanan-Khan A, Furman RR, et al. Acalabrutinib versus ibrutinib in previously treated chronic lymphocytic leukemia: results of the first randomized phase III trial. J Clin Oncol. 2021;39(31):3441–52. 10.1200/JCO.21.01210. 34310172 10.1200/JCO.21.01210 PMC8547923

[11] Omi A, Nomura F, Tsujioka S, Fujino A, Akizuki R. Efficacy and safety of ibrutinib in relapsed/refractory CLL and SLL in Japan: a post-marketing surveillance. J Clin Exp Hematop. 2022;62(3):136–46. 10.3960/jslrt.22002. 35831100 10.3960/jslrt.22002 PMC9635026

[12] Shyam Sunder S, Sharma UC, Pokharel S. Adverse effects of tyrosine kinase inhibitors in cancer therapy: pathophysiology, mechanisms and clinical management. Signal Transduct Target Ther. 2023;8(1):262. 10.1038/s41392-023-01469-6. 37414756 10.1038/s41392-023-01469-6 PMC10326056

[13] Iso H. Cardiovascular disease, a major global burden: epidemiology of stroke and ischemic heart disease in Japan. Glob Health Med. 2021;3(6):358–64. 10.35772/ghm.2020.01113. 35036616 10.35772/ghm.2020.01113 PMC8692094

[14] Linn BS, Linn MW, Gurel L. Cumulative illness rating scale. J Am Geriatr Soc. 1968;16(5):622–6. 10.1111/j.1532-5415.1968.tb02103.x. 5646906 10.1111/j.1532-5415.1968.tb02103.x

[15] Hallek M, Cheson BD, Catovsky D, Caligaris-Cappio F, Dighiero G, Dohner H, et al. iwCLL guidelines for diagnosis, indications for treatment, response assessment, and supportive management of CLL. Blood. 2018;131(25):2745–60. 10.1182/blood-2017-09-806398. 29540348 10.1182/blood-2017-09-806398

[16] Barr PM, Robak T, Owen C, Tedeschi A, Bairey O, Bartlett NL, et al. Sustained efficacy and detailed clinical follow-up of first-line ibrutinib treatment in older patients with chronic lymphocytic leukemia: extended phase 3 results from RESONATE-2. Haematologica. 2018;103(9):1502–10. 10.3324/haematol.2018.192328. 29880603 10.3324/haematol.2018.192328 PMC6119145

[17] Byrd JC, Hillmen P, O’Brien S, Barrientos JC, Reddy NM, Coutre S, et al. Long-term follow-up of the RESONATE phase 3 trial of ibrutinib vs ofatumumab. Blood. 2019;133(19):2031–42. 10.1182/blood-2018-08-870238. 30842083 10.1182/blood-2018-08-870238 PMC6509542

[18] Janssens A, Berneman ZN, Offner F, Snauwaert S, Mineur P, Vanstraelen G, et al. Effectiveness and safety of ibrutinib for chronic lymphocytic leukemia in routine clinical practice: 3-year follow-up of the Belgian ibrutinib Real-World Data (BiRD) study. Clin Hematol Int. 2022;4(4):133–43. 10.1007/s44228-022-00020-8. 36227519 10.1007/s44228-022-00020-8 PMC9763520

[19] Dartigeas C, Quinquenel A, Ysebaert L, Dilhuydy MS, Anglaret B, Slama B, et al. Final results on effectiveness and safety of ibrutinib in patients with chronic lymphocytic leukemia from the non-interventional FIRE study. Ann Hematol. 2024. 10.1007/s00277-024-05666-3. 38443660 10.1007/s00277-024-05666-3 PMC11971162

[20] Seymour JF, Byrd JC, Ghia P, Kater AP, Chanan-Khan A, Furman RR, et al. Detailed safety profile of acalabrutinib vs ibrutinib in previously treated chronic lymphocytic leukemia in the ELEVATE-RR trial. Blood. 2023;142(8):687–99. 10.1182/blood.2022018818. 37390310 10.1182/blood.2022018818 PMC10644206

[21] Kodani E, Atarashi H. Prevalence of atrial fibrillation in Asia and the world. J Arrhythmia. 2012;28(6):330–7. 10.1016/j.joa.2012.07.001.

[22] Ryan CE, Cheng MP, Issa NC, Brown JR, Davids MS. *Pneumocystis jirovecii* pneumonia and institutional prophylaxis practices in CLL patients treated with BTK inhibitors. Blood Adv. 2020;4(7):1458–63. 10.1182/bloodadvances.2020001678. 32282880 10.1182/bloodadvances.2020001678 PMC7160295

[23] Tey A, Schwarer J, Raffa R, Shi E, Paul E, Opat S, et al. High risk of infection in “real-world” patients receiving ibrutinib, idelalisib or venetoclax for mature B-cell leukaemia/lymphoma. Eur J Haematol. 2023;110(5):540–7. 10.1111/ejh.13928. 36656100 10.1111/ejh.13928 PMC10952205

[24] Kumar V, Ailawadhi S, Bojanini L, Mehta A, Biswas S, Sher T, et al. Trends in the risk of second primary malignancies among survivors of chronic lymphocytic leukemia. Blood Cancer J. 2019;9(10):75. 10.1038/s41408-019-0237-1. 31570695 10.1038/s41408-019-0237-1 PMC6768881

[25] Ogata D, Namikawa K, Nakano E, Fujimori M, Uchitomi Y, Higashi T, et al. Epidemiology of skin cancer based on Japan’s National Cancer Registry 2016–2017. Cancer Sci. 2023;114(7):2986–92. 10.1111/cas.15823. 37095610 10.1111/cas.15823 PMC10323082

