# Supplementary material for: Real-world effectiveness and safety of ibrutinib in patients with chronic lymphocytic leukemia in Japan: the Orbit study
Source: Int J Hematol. 2024 Nov 26;121(2):161–73. doi: 10.1007/s12185-024-03875-0 (PMC11782388; doi:10.1007/s12185-024-03875-0)
Supplement: Supplementary file 1 — Supplementary file1 (DOCX 96 KB) [file 12185_2024_3875_MOESM1_ESM.docx]

**Real-World Effectiveness and Safety of Ibrutinib in Patients with Chronic Lymphocytic Leukemia in Japan: the Orbit Study**

**Authors:** Tsuyoshi Muta^1^, Yosuke Masamoto^2^, Go Yamamoto^3^, Shingo Kurahashi^4^, Yoshihiro Kameoka^5,6^, Shuichi Ota^7^, Eri Matsuki^8^, Kazutaka Ozeki^9^, Takanori Toyama^10^, Naoki Takahashi^11^, Takahiro Kumode^12^, Nobuyuki Aotsuka^13^, Takuro Yoshimura^14^, Hideto Tamura^15,16^, Ai Omi^17^, Kazuhiro Shibayama^18^, Aki Watanabe^19^, Yasushi Isobe^20^, Kensuke Kojima^21^, Jun Takizawa^22^, Hirokazu Nagai^23^, Junji Suzumiya^24^, Sadao Aoki^25,26^

**Affiliations:** ^1^Department of Transfusion Medicine Clinical Laboratory, Hiroshima Red Cross Hospital and Atomic-bomb Survivors Hospital, Hiroshima, Japan; ^2^Department of Cell Therapy and Transplantation Medicine, The University of Tokyo Hospital, Tokyo, Japan; ^3^Department of Hematology, Toranomon Hospital, Tokyo, Japan; ^4^Department of Hematology and Oncology, Toyohashi Municipal Hospital, Aichi, Japan; ^5^Department of Hematology, Nephrology and Rheumatology, Akita University Graduate School of Medicine and Faculty of Medicine, Akita, Japan; ^6^Department of Hematology, Akita Red Cross Hospital, Akita, Japan; ^7^Department of Hematology, Sapporo Hokuyu Hospital, Sapporo, Japan; ^8^Division of Hematology, Department of Medicine Keio University School of Medicine, Tokyo, Japan; ^9^Department of Hematology and Oncology, JA Aichi Konan Kosei Hospital, Aichi, Japan; ^10^Department of Internal Medicine, Miyazaki Prefectural Nobeoka Hospital, Miyazaki, Japan; ^11^Department of Hematology, International Medical Center, Saitama Medical University, Saitama, Japan; ^12^Department of Hematology and Rheumatology, Kindai University Hospital, Osaka, Japan; ^13^Department of Hematology and Oncology, Japanese Red Cross Narita Hospital, Chiba, Japan; ^14^Department of Hematology, Osaka City General Hospital, Osaka, Japan; ^15^Department of Diabetes, Endocrinology and Hematology, Dokkyo Medical University Saitama Medical Center, Saitama, Japan; ^16^Department of Hematology, Nippon Medical School, Tokyo, Japan; ^17^Department of Medical Affairs, Janssen Pharmaceutical K.K., Tokyo, Japan; ^18^Statistics & Decision Sciences Japan, Janssen Pharmaceutical K.K., Tokyo, Japan; ^19^Medical Affairs Delivery Unit, Global Development, Janssen Pharmaceutical K.K., Tokyo, Japan; ^20^Department of Medical Oncology, Hematology and Infectious Diseases, Fukuoka University Hospital, Fukuoka, Japan; ^21^Department of Hematology, Kochi Medical School, Kochi University, Kochi, Japan; ^22^Department of Hematology, Endocrinology and Metabolism, Niigata University Faculty of Medicine, Niigata, Japan; ^23^Hematology Department, National Hospital Organization Nagoya Medical Center, Aichi, Japan; ^24^Department of Hematology, Koga Community Hospital, Yaizu, Japan; ^25^Department of Medical Technology, Faculty of Medical Technology, Niigata University of Pharmacy and Medical and Life Sciences, Niigata, Japan; ^26^Department of Hematology, Niigata Minami Hospital, Niigata, Japan.

# Supplementary Material

## Supplementary Table S1.

Prior treatment for chronic lymphocytic leukemia in patients with relapsed or refractory disease (n=94).

|  | **N=94** |
| --- | --- |
| No. of prior treatment lines |  |
| Median (range) | 2 (1–6) |
| Category, n (%) |  |
| 1 | 44 (46.8) |
| 2 | 22 (23.4) |
| ≥3 | 23 (24.5) |
| Unknown | 5 (5.3) |
| Most recent CLL treatment, n (%) |  |
| Fludarabine | 22 (23.4) |
| Cyclophosphamide | 13 (13.8) |
| Bendamustine + rituximab | 10 (10.6) |
| Rituximab | 9 (9.6) |
| Fludarabine + cyclophosphamide + rituximab | 8 (8.5) |
| Bendamustine | 5 (5.3) |
| Fludarabine + rituximab | 5 (5.3) |
| Ofatumumab | 4 (4.3) |
| R-CHOP | 3 (3.2) |
| Fludarabine + cyclophosphamide | 2 (2.1) |
| Cyclophosphamide + corticosteroid | 2 (2.1) |
| Cyclophosphamide + corticosteroid + procarbazine | 2 (2.1) |
| Cyclophosphamide + corticosteroid + pirarubicin + vincristine | 1 (1.1) |
| Cyclophosphamide + corticosteroid + procarbazine + etoposide | 1 (1.1) |
| Cyclophosphamide + corticosteroid + vincristine | 1 (1.1) |
| Rituximab + cyclophosphamide + corticosteroid | 1 (1.1) |
| Rituximab + cyclophosphamide + corticosteroid + etoposide + procarbazine | 1 (1.1) |
| Rituximab + cyclophosphamide + vincristine + prednisone | 1 (1.1) |
| Venetoclax | 1 (1.1) |
| Yttrium ibritumomab tiuxetan | 1 (1.1) |
| Unknown | 1 (1.1) |

CLL, chronic lymphocytic leukemia; R-CHOP, rituximab, cyclophosphamide, doxorubicin, vincristine, and prednisone.

## Supplementary Table S2.

Baseline laboratory values in the safety analysis set.

| **Laboratory values, median (range)** | **Total population**  **n=237^a^** | **1L CLL**  **n=142** | **RR CLL**  **n=94** |
| --- | --- | --- | --- |
| IgA, mg/dL | 74.0 (7.0–3769.0) | 79.6 (11.0–3769.0) | 70.0 (7.0–998.0) |
| IgG, mg/dL | 830.5 (34.0–5,912.6) | 946.0 (265.0–5,912.6) | 626.0 (34.0–2,141.0) |
| IgM, mg/dL | 29.0 (2.0–2,279.2) | 39.0 (8.0–1,569.0) | 26.0 (2.0–2,279.2) |
| WBC count, /μL | 18,700 (1,239–384,800) | 20,200 (1,239–384,800) | 16,310 (1,870–286,000) |
| Neutrophil count, /μL | 2,918.7 (0.0–22,072.0) | 3,068.5 (0.0–22,072.0) | 2,573.0 (0.0–13,040.0) |

^a^Includes one patient with unknown CLL/SLL status, who met exclusion criteria immediately after registration and had no data collected thereafter.

1L, first-line; CLL, chronic lymphocytic leukemia; IgA/G/M, immunoglobulin A/G/M; RR, relapsed or refractory; WBC, white blood cell.

## Supplementary Table S3.

Summary of subsequent treatment after ibrutinib in the per-protocol set.

|  | **1L CLL**  **n=141** | **RR CLL**  **n=93** |
| --- | --- | --- |
| Post-ibrutinib treatment, n (%) |  |  |
| Yes | 31 (22.0) | 31 (33.3) |
| No | 109 (77.3) | 62 (66.7) |
| Unknown | 1 (0.7) | 0 |
| Regimen, n (%) | n=31 | n=31 |
| Venetoclax ± rituximab | 12 (38.7) | 14 (45.2) |
| Acalabrutinib | 10 (32.3) | 2 (6.5) |
| R-CHOP | 2 (6.5) | 3 (9.7) |
| Bendamustine + rituximab | 2 (6.5) | 1 (3.2) |
| Corticosteroid | 2 (6.5) | 1 (3.2) |
| Doxorubicin + bleomycin + vinblastine + dacarbazine | 1 (3.2) | 0 |
| Etoposide | 1 (3.2) | 0 |
| Rituximab | 1 (3.2) | 0 |
| Cyclophosphamide | 0 | 4 (12.9) |
| Fludarabine | 0 | 2 (6.5) |
| Bendamustine | 0 | 1 (3.2) |
| Cyclophosphamide + corticosteroid | 0 | 1 (3.2) |
| Hydroxycarbamide | 0 | 1 (3.2) |
| Ofatumumab | 0 | 1 (3.2) |

1L, first-line; CLL, chronic lymphocytic leukemia; R-CHOP, rituximab, cyclophosphamide, doxorubicin, vincristine, and prednisone; RR, relapsed or refractory.

## Supplementary Figure S1.

Study design.


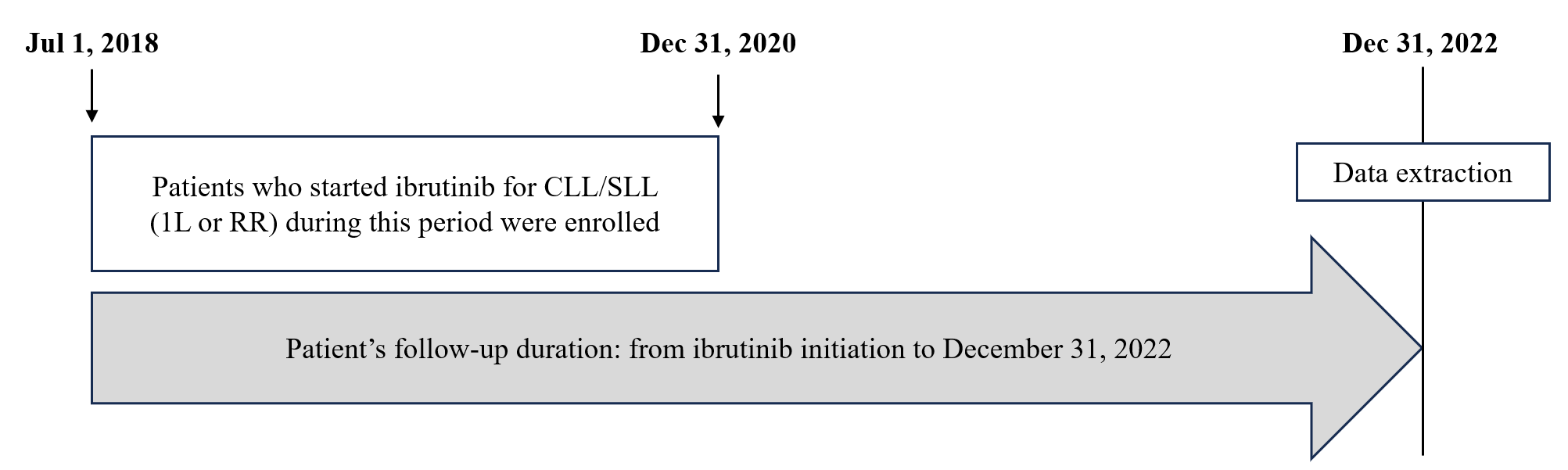


1L, first-line; CLL, chronic lymphocytic leukemia; RR, relapsed or refractory; SLL, small lymphocytic lymphoma.

## Supplementary Figure S2.

Kaplan–Meier curves of time to next treatment in patients with chronic lymphocytic leukemia receiving ibrutinib as (a) first-line therapy and (b) for relapsed or refractory disease in the per-protocol set.


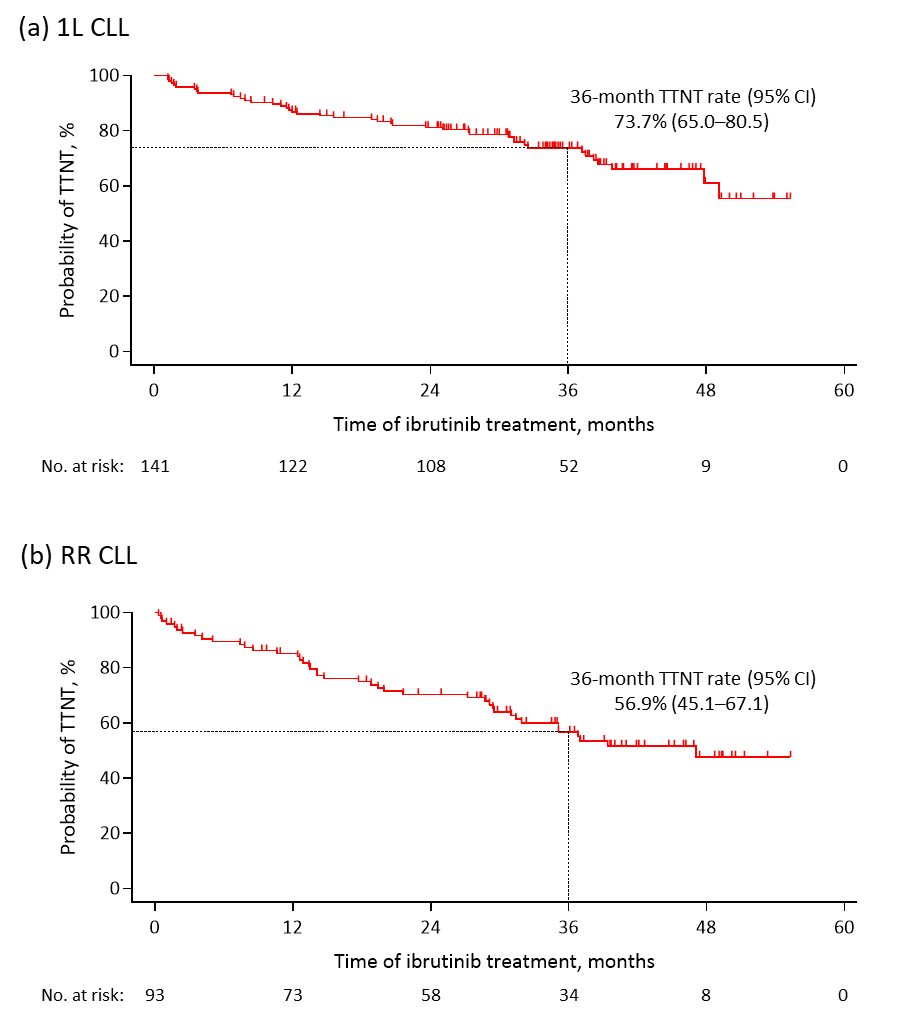


1L, first-line; CI, confidence interval; CLL, chronic lymphocytic leukemia; No., number; RR, relapsed or refractory; TTNT, time to next treatment.
